# Long Non-coding RNAs Involved in Metabolic Alterations in Breast and Prostate Cancers

**DOI:** 10.3389/fonc.2020.593200

**Published:** 2020-10-06

**Authors:** Shuhei Kamada, Toshihiko Takeiwa, Kazuhiro Ikeda, Kuniko Horie-Inoue, Satoshi Inoue

**Affiliations:** ^1^Division of Systems Medicine and Gene Therapy, Saitama Medical University, Saitama, Japan; ^2^Department of Urology, Graduate School of Medicine, Chiba University, Chiba, Japan; ^3^Department of Systems Aging Science and Medicine, Tokyo Metropolitan Institute of Gerontology, Tokyo, Japan

**Keywords:** long non-coding RNA, cancer metabolism, glucose, oxidative phosphorylation, lipid, breast cancer, prostate cancer

## Abstract

Breast and prostate cancers are the most prevalent cancers in females and males, respectively. These cancers exhibit sex hormone dependence and thus, hormonal therapies are used to treat these cancers. However, acquired resistance to hormone therapies is a major clinical problem. In addition, certain portions of these cancers initially exhibit hormone-independence due to the absence of sex hormone receptors. Therefore, precise and profound understanding of the cancer pathophysiology is required to develop novel clinical strategies against breast and prostate cancers. Metabolic reprogramming is currently recognized as one of the hallmarks of cancer, as exemplified by the alteration of glucose metabolism, oxidative phosphorylation, and lipid metabolism. Dysregulation of metabolic enzymes and their regulators such as kinases, transcription factors, and other signaling molecules contributes to metabolic alteration in cancer. Moreover, accumulating lines of evidence reveal that long non-coding RNAs (lncRNAs) regulate cancer development and progression by modulating metabolism. Understanding the mechanism and function of lncRNAs associated with cancer-specific metabolic alteration will therefore provide new knowledge for cancer diagnosis and treatment. This review provides an overview of recent studies regarding the role of lncRNAs in metabolism in breast and prostate cancers, with a focus on both sex hormone-dependent and -independent pathways.

## Breast and Prostate Cancers

In developed countries, breast and prostate cancers are the most common malignancies in women and men, respectively (1). In both breast and prostate cancers, growth and survival are mainly controlled by sex steroid hormones, which include estrogens and androgens, respectively (2). These unique properties have been targeted in treating breast and prostate cancers with hormone therapy (3). However, during long-term hormone therapy, acquired resistance develops in many patients (4), and there are few effective treatments for hormone-refractory cancers. In addition, from the tumor diagnosis, breast cancers negative for estrogen receptor α (ERα) such as triple-negative breast cancer (TNBC) exhibit no response to hormone therapy (5). In the case of prostate cancer, most cancers are initially androgen-dependent and respond to hormone therapies; however, they eventually acquire endocrine therapy resistance and become castration-resistant prostate cancer (CRPC) (6). Thus, understanding both mechanisms of hormone dependence and hormone resistance is important for the development of diagnosis and treatment options of breast and prostate cancers.

## Long Non-Coding RNA (lncRNA)

**FIGURE 1:**
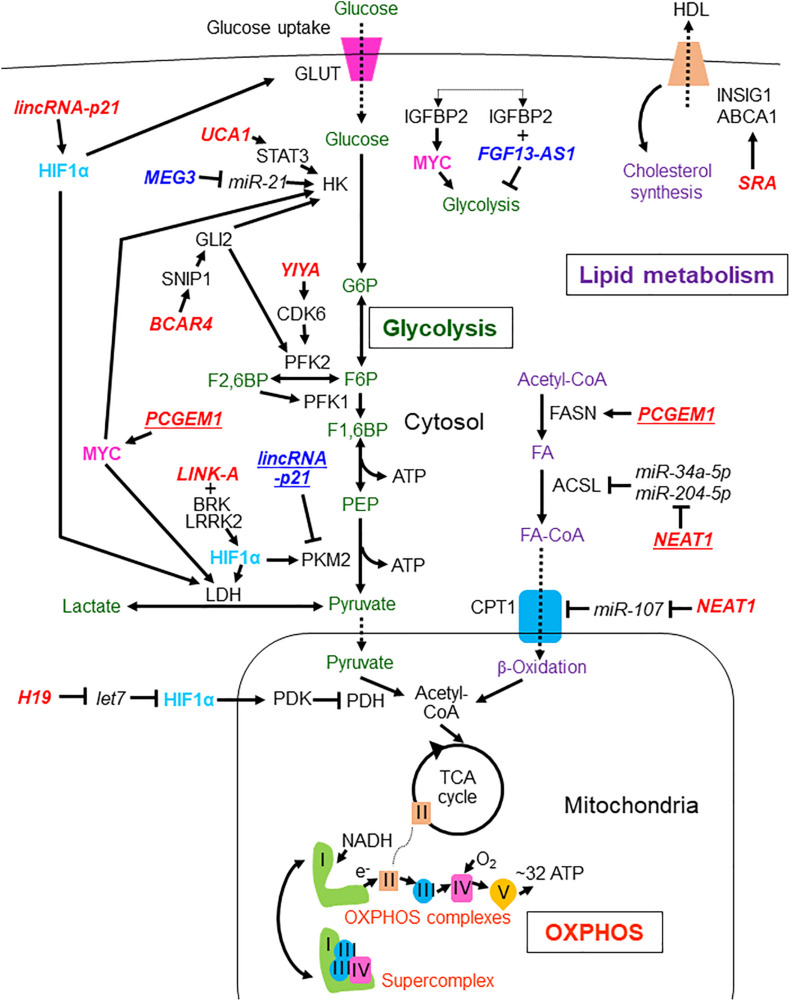
LncRNAs involved in the regulation of glycolysis, OXPHOS, and lipid metabolism in breast and prostate cancers. LncRNAs reported in breast and prostate cancers are shown without and with underlines, respectively. Tumor suppressive lncRNAs are indicated by dark blue characters while oncogenic lncRNAs are shown with red characters. The metabolites and processes of glycolysis, oxidative phosphorylation (OXPHOS), and lipid metabolism are shown with green, orange, and purple characters, respectively. Transcription factors HIF1α and MYC are presented with light blue and pink characters, respectively. lincRNA-p21, long intergenic non-coding RNA-p21;UCA1, urothelial carcinoma associated 1; MEG3, maternally expressed 3; FGF13-AS1, FGF13 antisense RNA 1; BCAR4, breast cancer anti-estrogen resistance 4; LINK-A, long intergenic non-coding RNA for kinase activation; SRA, steroid receptor RNA activator; PCGEM1, prostate cancer gene expression marker 1; NEAT1, nuclear enriched abundant transcript 1; GLUT, glucose transporter; HK, hexokinase; G6P, glucose-6-phosphate; PFK2, phosphofructokinase-2/fructose bisphosphatase-2; F2,6BP, fructose 2,6-bisphosphate; PFK1, phosphofructokinase-1; F1,6BP, fructose 1,6-bisphosphate; PEP, phosphoenolpyruvate; PKM2, pyruvate kinase isozymes M2; LDH, lactate dehydrogenase; HDL, high-density lipoprotein; INSIG1, insulin-induced gene 1; ABCA1, ATP binding cassette subfamily A member 1; FASN, fatty acid synthase; ACSL, Acyl-CoA synthetase long chain family; FA, fatty acid; CPT1, carnitine palmitoyltransferase 1; PDH, pyruvate dehydrogenase; TCA, tricarboxylic acid; OXPHOS, oxidative phosphorylation; STAT3, signal transducer and activator of transcription 3; HIF1α, hypoxia-inducible factor 1A; GLI2, glioma-associated oncogene homolog 2; SNIP1, Smad nuclear-interacting protein 1; CDK6, cyclin-dependent kinase 6; MYC, MYC proto-oncogene; BRK, breast tumor kinase; LRRK2, leucine rich-repeat kinase 2; PDK, pyruvate dehydrogenase kinase; Plus (+) means binding of lncRNA to protein.

Recent cancer research has focused on the roles and importance of lncRNAs in cancer development. Although up to 70% of the human genome is actively transcribed, only 2% of transcripts are translated into proteins (7). Transcripts that do not encode proteins are called non-coding RNAs, among them those with a length >200 nucleotides are categorized as lncRNAs (8). LncRNAs exert their function by regulating a variety of intracellular processes, for example, (1) recruiting epigenetic modifiers and transcription factors to the enhancer/promoter of the lncRNA target gene, (2) forming RNA-RNA binding with its target mRNA for mRNA degradation, (3) acting as a decoy, enabling avoidance of the associated protein from transcriptional regulatory regions, and (4) acting as a molecular sponge or as a competitive endogenous RNA (ceRNA) for microRNA (4, 9–11). In terms of the contribution of lncRNAs to cancer metabolism, characterization of lncRNAs associated with glucose metabolism has proceeded (12, 13) probably because the Warburg effect with aerobic glycolysis has been considered as a central event for cancer metabolism. Recently, lncRNA functions in other metabolic pathways including mitochondrial oxidative phosphorylation (OXPHOS) and lipid biosynthesis have been reported. Moreover, it has been shown that lncRNAs also modulate hormone sensitivity and resistance in breast and prostate cancers (4, 10, 14). These findings suggest that the metabolism-associated lncRNAs would be new therapeutic targets for hormone-refractory breast and prostate cancers. Further intensive analyses will reveal the precise characteristics of these lncRNAs including their expression profiles, binding factors, and three-dimensional structures, using advanced technologies such as high-throughput sequencing, mass spectrometry, and bioinformatics. Based on the molecular aspects of lncRNAs, their expressions and functions could be modulated by nucleic acid therapy using antisense oligonucleotide and RNA interference technique, as well as by small-molecule drugs that bind to lncRNAs. Moreover, effector proteins of lncRNAs will be also potential targets for modulating the lncRNA-associated signaling (4, 7). In addition, lncRNA delivery and lncRNA-inducing drugs should be important to modify the functions of these lncRNAs. While efforts are underway to improve the molecular technology and skills for analyzing lncRNAs, a comprehensive understanding for their roles in cancer metabolism remains to be elusive and further investigation will be required to apply these molecules for cancer therapy. In the present review, we provide an overview of the recent findings regarding lncRNA involvement in cancer metabolism, with a focus on breast and prostate cancers (Figure 1).

## Glucose Metabolism and lncRNA

Metabolic reprogramming is one of the hallmarks of cancer. Tumor cells preferentially use glycolysis for ATP production even in the presence of oxygen (15). However, the ATP production efficiency of glycolysis itself is not high compared with that of OXPHOS. Glycolysis-dependent cancer cells will be prone to undergo metabolic reprogramming as they eventually need to compensate for lower energy production efficiency by glycolysis due to high turnover of cell proliferation. As one strategy for the improvement of energy production efficiency, cancer cells often facilitate glucose uptake and usage by upregulating glucose transporters and glycolytic enzymes (16). For instance, the glucose transporter (GLUT) family, which is responsible for cellular glucose uptake, is upregulated in multiple cancer types (17). Hexokinases (HK), which convert glucose to glucose-6-phosphate (G6P) at the first step of glycolysis, are particularly activated in cancer cells wherein they function as a driver of glycolysis (18). Phosphofructokinase-2/fructose-bisphosphatase-2 (PFK2), which is responsible for the synthesis of fructose-2,6-bisphosphate, is also upregulated in various cancers (19). Pyruvate kinase catalyzes the final step of glycolysis to produce pyruvate (20), and pyruvate kinase M2 (PKM2) among the 4 pyruvate kinase isomers is assumed to play a potential role in tumorigenesis of several cancers including breast (21) and prostate cancer (22). Pyruvate dehydrogenase kinase (PDK) phosphorylates and inactivates the pyruvate dehydrogenase complex, which synthesizes acetyl coenzyme A as a critical substrate of tricarboxylic acid (TCA) cycle from pyruvate. Inhibition of pyruvate dehydrogenase complex activity by PDK-mediated phosphorylation is involved in cancer pathophysiology (23–25). Lactate dehydrogenase A (LDHA) is an enzyme that converts pyruvate to lactate, and its overexpression is also observed in many types of cancers (26). Oncogenes such as MYC proto-oncogene (MYC) and hypoxia-inducible factor 1A (HIF1α) have been also shown as master regulators for glucose metabolic alteration in cancer cells (27, 28). For example, the expression of glycolytic enzymes such as HK2, PFK, and LDHA is cooperatively regulated by MYC and HIF1α (29–31). In the following sections, we will discuss several major lncRNAs involved in the metabolic pathways in breast and prostate cancers.

### H19

*H19* is an lncRNA that regulates glucose metabolism in both breast and prostate cancers. In breast cancer, *H19* acts as a ceRNA that sequesters microRNA *let-7*, leading to the upregulation of the *let-7* target gene *HIF1A* that encodes HIF1α protein. This resulting HIF1α upregulation leads to an increase in *PDK1* expression (32). PDK1 is the most dominant subtype of the PDK family that is involved in breast cancer progression and metastasis (32–34). Interestingly, hormone therapy drugs such as tamoxifen or fulvestrant elevate *H19* expression in ERα-positive breast cancer cells, which contributes to the acquired resistance to these drugs (35). In addition, *H19* depletion decreases the growth and the glucose and lactate levels in prostate cancer DU145 and PC-3 cells whereas the mechanism remains to be clarified (36).

### Long Intergenic Non-coding RNA-p21 (*lincRNA-p21*)

*LincRNA-p21* is inducible by hypoxia or HIF1α function in breast cancer cells (4), and binds to HIF1α and its E3 ubiquitin ligase von Hippel-Lindau (VHL), thereby disrupting the VHL-HIF1α interaction. This dissociation attenuates VHL-mediated HIF1α ubiquitination and results in HIF1α stabilization as a positive feedback loop for HIF1α-dependent pathways. It is revealed that *lincRNA-p21* promotes hypoxia-induced lactate production and glucose uptake in breast cancer cells through the upregulation of *GLUT1* and *LDHA* (4). In contrast, *lincRNA-p21* is often downregulated in prostate cancer, and *lincRNA-p21* silencing activates PKM2 and glycolysis in LNCaP and DU145 prostate cancer cells (37). The differential effects of *lincRNA-p21* on glycolysis may be attributed to a cancer type-specific metabolic pathway regulation between breast and prostate cancers.

### Long Intergenic Non-coding RNA for Kinase Activation (*LINK-A*)

*LINK-A* has been identified as a cytoplasmic lncRNA that is preferentially expressed in TNBC (38). *LINK-A* binds with both breast tumor kinase (BRK) and leucine rich-repeat kinase 2 (LRRK2), and this process is stimulated by the signaling from heparin-binding epidermal growth factor (HB-EGF)-triggered epidermal growth factor receptor (EGFR) and transmembrane glycoprotein non-metastatic melanoma protein B (GPNMB) complex formation. BRK phosphorylates the Tyr 565 of HIF1α, which interferes with the Pro 564 hydroxylation-mediated HIF1α degradation, whereas LRRK2 phosphorylates HIF1α Ser 797 which enhances HIF1α transcriptional activity. These HIF1α phosphorylation events facilitate glycolysis reprogramming, i.e., glucose uptake and lactate production, in TNBC (38).

### Prostate Cancer Gene Expression Marker 1 (*PCGEM1*)

*PCGEM1* was originally identified as an androgen-induced prostate-specific lncRNA. Its overexpression is highly associated with prostate tumors, and it promotes cancer cell proliferation (39, 40). In addition to hormonal regulation, *PCGEM1* functions as a coactivator of MYC and is estimated to induce expression of glycolytic enzymes such as HK2, glucose-6-phosphate dehydrogenase (G6PD), and LDHA (41). *PCGEM1* overexpression increases glucose uptake and lactate production along with the pentose phosphate shunt to provide a sufficient supply of nucleotides and lipids in prostate cancer.

### FGF13 Antisense RNA 1 (*FGF13-AS1*)

*FGF13-AS1* binds with an RNA-binding protein, insulin-like growth factor 2 mRNA binding protein (IGF2BP), which interacts with the *MYC* mRNA (42). Binding of *FGF13-AS1* with IGF2BP disrupts the interaction between *IGF2BP* and *MYC* mRNAs, leading to a decrease in the half-life of *MYC* mRNA. *FGF13-AS1* overexpression decreases glucose consumption and lactate production in TNBC subtype MDA-MB-231 cells, while *FGF13-AS1* depletion increases them in luminal subtype MCF-7 cells. These results indicate that *FGF13-AS1* suppresses the glucose metabolism in these breast cancer subtypes. *FGF13-AS1* also suppresses the spheroid formation and stemness properties of these breast cancer subtypes (42).

### Urothelial Carcinoma Associated 1 (*UCA1*)

*UCA1* participates in glucose metabolism in several cancer types (43–46). Particularly, in tumor suppressor Merlin-deficient breast cancer cells, upregulated *UCA1* stimulates glucose consumption and lactate production through the activation of serine/threonine kinase AKT and signal transducer and activator of transcription 3 (STAT3) with a simultaneous increase in HK2 expression (47). Moreover, *UCA1* has been implicated in hormone therapy resistance in breast cancer cells as it sponges *miR-18a*, leading to HIF1α activation or inhibition of mTOR signaling (48, 49), suggesting its multiple functions in breast cancer metabolism.

### Maternally Expressed 3 (*MEG3*)

As shown in other type of cancer (50), the lncRNA *MEG3* is downregulated in breast cancer tissues (51). *MEG3* acts as a molecular sponge of *miR-21*, resulting in the repression of HK2 protein levels and the glycolytic pathway (i.e., glucose consumption and lactate production) in both MCF-7 and MDA-MB-231 cells (51).

### YIYA

*YIYA* is an lncRNA expressed in approximately 40% of clinical breast cancer tumors and is associated with poor recurrence-free survival of the patients (52). This report also indicates that *YIYA* binds with cyclin-dependent kinase 6 (CDK6) to enhance CDK6-dependent phosphorylation of PFK2. In breast cancer cells, *YIYA* stimulates cell proliferation with an increase in the glycolytic pathway (i.e., elevation of glucose consumption and lactate production) (52).

### Breast Cancer Anti-Estrogen Resistance 4 (*BCAR4*)

*BCAR4* has been identified as a TNBC-upregulated lncRNA that is essential for breast cancer metastasis (53). This report indicates that *BCAR4* associates with Smad nuclear-interacting protein 1 (SNIP1) and serine/threonine-protein phosphatase 1 regulatory subunit 10 (PPP1R10/PNUTS) to promote the transcriptional signaling by non-canonical hedgehog signal mediator glioma-associated oncogene homolog 2 (GLI2) by discharging the inhibitory role of SNIP1 on p300 histone acetyltransferase activity. More recently, it was demonstrated that *BCAR4* is upregulated by the Hippo pathway downstream effector, Yes-associated protein (YAP), and modulates Hedgehog signaling to activate transcription of HK2 and PFK2 in TNBC cells (54). Notably, *BCAR4* induces glucose uptake and lactate production in these cells. Furthermore, high expression of both *BCAR4* and YAP is associated with poor survival of patients with breast cancer, suggesting a critical role for the YAP-*BCAR4*-glycolysis axis in this disease.

## OXPHOS With lncRNA

As aerobic glycolysis has been assumed as a major energy resource for cancer metabolism, mitochondrial OXPHOS has been rather considered as a second-grade metabolic pathway in cancers. Nevertheless, recent lines of evidence indicate that OXPHOS is also crucial for energy metabolism in cancers (55). The OXPHOS system comprises five enzyme complexes embedded in the inner mitochondrial membrane: complex I (NADH dehydrogenase), complex II (succinate ubiquinone oxidoreductase), complex III (ubiquinol cytochrome *c* oxidoreductase), complex IV (cytochrome *c* oxidoreductase), and complex V (ATP synthase). While the contributions of glycolysis and OXPHOS to ATP biosynthesis vary among cell types as shown by a meta-analysis, the contribution ratios of OXPHOS were almost equally ∼80% in both normal cells and cancer cells (56). Moreover, it has been revealed that OXPHOS rather remains the most significant source of energy production in tumors, suggesting that mitochondrial respiration is not generally impaired in cancer cells (57).

Several studies indicate that OXPHOS expression and function are both upregulated in breast and prostate cancers. Upregulation of OXPHOS gene expression and enzyme activity is detected in patients with breast cancer (58). In tumor suppressor RB1-deficient TNBC, OXPHOS is highly upregulated to promote cancer cell proliferation (59, 60). Moreover, cytochrome *c* oxidase subunit 7a-related polypeptide (COX7RP), which was originally identified as an estrogen-responsive gene, promotes mitochondrial respiration and cell proliferation in breast cancer cells by accelerating the formation of mitochondrial supercomplexes, which contributes to efficient ATP synthesis by assembling complexes I, III, and IV (61).

It has been recently shown that a compound-induced mitochondrial fragmentation leading to the disruption of OXPHOS and ATP synthesis inhibits the proliferation of prostate cancer PC3 cells (62). An integrated analysis of RNA-sequencing datasets and proteome data indicates that OXPHOS-related genes are upregulated in prostate cancer and negatively associated with STAT3 expression (63). Another integrated analysis elucidates a preservative role of MYC on OXPHOS function in prostate cancer tumorigenesis (64). In this report, MYC knockdown in PC3 cells reduced the levels of oxidative phosphorylation and TCA cycle metabolites along with decreased oxygen consumption rates (OCR), leading to diminished ATP production. Docetaxel-resistant PC3 cells show a metabolic shift from glycolysis toward mitochondrial respiration (65). Interestingly, RNA-seq analysis of prostate tissues shows remarkable enrichment of OXPHOS-related genes in CRPC (66). Moreover, a study analyzing substrate-specific OXPHOS capacities using primary human prostate tissues revealed that the malignant tissues exhibits a significant metabolic shift toward higher succinate oxidation by complex II, particularly in high-grade prostate tumors (67).

OXPHOS upregulation, however, is rather limited to particular cancer subtypes (68–70). In addition, OXPHOS activity can be coordinated by many factors including mitochondrial DNA content, expression levels of respiration complexes subunits and regulatory genes, and the cellular environment. Up to date, few studies have been performed for the analysis of OXPHOS activity in the association with lncRNAs. Further analysis will clarify the role of lncRNA on OXPHOS in different cancer types, including breast and prostate cancers.

Integrated analysis of the mitochondrial proteome and the gene expression dataset showed that the lncRNA *small nucleolar RNA host gene 3* (*SNHG3*) is related to ovarian cancer survival as well as the expression of energy metabolism-related genes such as complex III subunit ubiquinol-cytochrome *c* reductase hinge protein (UQCRH) (71). *In silico* analysis speculates that *SNHG3* can function by sponging miR-186-5p. In gastric cancer tissues, expression levels of lncRNA *MIF-AS1* (*lncMIF-AS1*) and complex IV subunit NADH dehydrogenase1 alpha subcomplex 4 (NDUFA4) are higher compared with those in non-cancerous tissues (72). Mechanistically, *lncMIF-AS1* sponges *miR-212-5p* to activate NDUFA4 expression. *lncMIF-AS1* overexpression promotes proliferation and decreases apoptosis of gastric cancer cells through the activation of oxygen consumption and ATP production. The lncRNA *cytoplasmic endogenous regulator of oxidative phosphorylation 1* (*Cerox1*) sponges *miR-488-3p* and elevates the expression of several subunits of complex I in mouse neuroblastoma cells (73).

## Lipid Metabolism With lncRNA

Altered regulation in the synthesis and utilization of lipids is also a hallmark of cancers as it meets the high energy demand required for cancer proliferation and survival. In contrast to normal tissues and cells, malignant tumors prefer to synthesize lipids through the *de novo* pathway (74, 75). Increased expression or abnormal activity of key lipogenic enzymes such as fatty acid synthase (FASN) and acetyl coenzyme A (acetyl-CoA) carboxylase is often attributed to the high growth rate and lipogenic phenotype of tumor cells (76–80). Moreover, carnitine palmitoyl transferase (CPT) 1 and 2, which are rate-limiting enzymes involved in mitochondrial fatty acid transportation, play crucial roles in increasing fatty acid oxidation required for the cellular fuel demands of breast cancer cells (81). CPT1A/CPT2 were highly expressed in recurrent human breast cancers and are associated with poor prognosis. Moreover, increased fatty acid oxidation has also been suggested as a source of energy (82), along with increased steroidogenesis by upregulation of Acyl-CoA synthetase long-chain family member (ACSL) 3 (ACSL3) in prostate cancer (83). ACSL4, as well as ACSL3, which is an enzyme that converts fatty acids to acyl-CoA, is also involved in the loss of androgen sensitivity and acquisition of castration resistance, leading to cancer growth and invasion in prostate cancer (83–85). We mention several lncRNAs involved in breast and prostate cancers in the following sections.

### Nuclear Enriched Abundant Transcript 1 (*NEAT1*)

In breast and prostate cancers, *NEAT1* stimulates the TCA cycle by promoting the use of free fatty acids as fuel. *NEAT1* upregulates *CPT1A* expression by inhibiting *miR-107* to promote the progression of breast cancer cells (86). CPT1A synthesizes acylcarnitines, which are transported from the cytosol into the mitochondria, and their acyl groups are metabolized through the TCA cycle. *NEAT1* also affects *ACSL4* expression by competitively sponging both *miR-34a-5p* and *miR-204-5p* in prostate cancer (87). The alteration of *ACSL4* expression is essential in the development and progression of breast and prostate cancers: *ER* expression is inversely correlated with *ACSL4* expression in breast cancer (84), and ACSL4 is also involved in the loss of steroid hormone sensitivity and the acquisition of castration resistance in prostate cancer (85, 88). Recent findings indicate that *NEAT1* affects mitochondrial dynamics and functions by regulating the sequestration of mRNAs encoding mitochondrial proteins in nuclear bodies called paraspeckles (89, 90).

### PCGEM1

In addition to regulating glucose metabolism as described above, *PCGEM1* widely regulates metabolic gene expression, including lipid metabolism in prostate cancer cells (41). The expression levels of several enzymes involved in lipid biosynthesis, such as those of FASN and acetyl-CoA carboxylase alpha (ACACA), are decreased by *PCGEM1* knockdown. *PCGEM1*-overexpressing prostate cancer cells showed an increase in the cellular level of citrate, indicating enhanced fatty acid synthesis.

### Steroid Receptor RNA Activator (*SRA*)

*SRA* was originally identified as a lncRNA that coactivates steroid hormone receptor transcriptional activity by associating with steroid receptor coactivator-containing complexes (91). Furthermore, *SRA* has also been revealed to interact with other transcription factors (92, 93). *SRA* overexpression has been found in various tumors including prostate cancer (91, 94). Both androgen receptor-dependent and -independent mechanisms are involved in *SRA-*mediated prostate cancer progression (95, 96). *SRA* expression is also significantly upregulated in breast cancer tissues (97). In MCF-7 breast cancer cells, *SRA* silencing decreased mRNA levels of *insulin-induced gene 1 protein* (*INSIG1)* and cholesterol transporter ATP-binding cassette transporter *ABCA1*, suggesting its role in lipids/cholesterol homeostasis (98, 99).

## Conclusion

In this review, we summarized the functions and mechanisms of cancer metabolism-related lncRNAs especially in breast and prostate cancers (Table 1). We consider that these lncRNA functions may play critical roles in cancer pathophysiology from the viewpoint of their contribution to metabolic reprogramming. Moreover, the functions of cancer metabolism-related lncRNAs will be modulated by hormone status in breast and prostate cancers. Elucidating the mechanisms underlying metabolic alterations regulated by lncRNAs will lead to the development of new diagnostic and therapeutic options for both breast and prostate cancers.

**TABLE 1:** LncRNAs involved in breast and prostate cancer metabolism.

LncRNA	Chr	Length (Kb)	Cancer	Oncogenic/suppressive	Mechanism/target/action
**Glucose metabolism**					
*H19*	11p15.5	2.3	BC	Oncogenic	Sponges *let-7* leading to activation of HIF1α and PDK1 (33)
			PC	Oncogenic	Increases glucose and lactate levels (36)
*lincRNA-p21*	6p21.2	3.1	BC	Oncogenic	Binds to and stabilizes HIF1α, and upregulates GLUT1 and LDHA (12)
			PC	Suppressive	Downregulates *PKM2* (34)
*LINK-A*	1q43	1.5	BC	Oncogenic	Binds to BRK and LRRK2 to facilitate phosphorylation and activation of HIF1α (35)
*PCGEM1*	2q32.3	0.4	PC	Oncogenic	Functions as cofactor of MYC to activate glycolytic enzymes such as HK2, G6PD, and LDHA (38)
*FGF13-AS1*	Xq26.3	0.8	BC	Suppressive	Downregulates MYC by competitive binding with IGF2BP and inhibits glycolysis (39)
*UCA1*	19p13.12	2.3	BC	Oncogenic	Activates AKT and STAT3, and increases HK2 (44)
*MEG3*	14q32.2	1.6	BC	Suppressive	Sponges miR-21, leading to HK2 reppression (48)
*YIYA*	1q32.3	1.9	BC	Oncogenic	Binds with CDK6, leading to phosphorylation of PFK2 (49)
*BCAR4*	16p13.13	1.3	BC	Oncogenic	Binds with SNIP1 and PNUTS to activate HK2 and PFK2 expression by releasing p300 inactivation (50, 51)
**Lipid metabolism**					
*NEAT1*	11q13.1	Short: 3.7, long: 23	BC	Oncogenic	Sponges *miR-107*, leading to *CPT1A* expression (83)
			PC	Oncogenic	Sponges *miR-34a-5p* and *miR-204-5p*, leading to *ACSL4* expression (84)
*PCGEM1*	2q32.3	0.4	PC	Oncogenic	Functions as cofactor of MYC to activate lipid biosynthesis genes such as FASN and ACACA (38)
*SRA*	5q31.1	0.7	BC	Oncogenic	Interacts with steroid receptor coactivator complexes (95, 96)
			PC	Oncogenic	

*Chr, chromosome; BC, breast cancer; PC, prostate cancer.*

## Author Contributions

SK, TT, KI, KH-I, and SI: conception, providing the data and design. SK and TT: manuscript writing. KI, KH-I, and SI: conception and final approval of manuscript. All authors contributed to the article and approved the submitted version.

## Conflict of Interest

The authors declare that the research was conducted in the absence of any commercial or financial relationships that could be construed as a potential conflict of interest.
